# Using multicriteria decision analysis during drug development to predict reimbursement decisions

**DOI:** 10.3402/jmahp.v2.25270

**Published:** 2014-10-31

**Authors:** Paul Williams, Josephine Mauskopf, Jake Lebiecki, Anne Kilburg

**Affiliations:** ^a^ RTI Health SolutionsDurham, NC, USA; ^b^ RTI Health SolutionsManchester, UK

**Keywords:** multicriteria decision analysis, Spain, United Kingdom, Germany, reimbursement, additional benefit, cost-effectiveness

## Abstract

**Background:**

Pharmaceutical companies design clinical development programs to generate the data that they believe will support reimbursement for the experimental compound.

**Objective:**

The objective of the study was to present a process for using multicriteria decision analysis (MCDA) by a pharmaceutical company to estimate the probability of a positive recommendation for reimbursement for a new drug given drug and environmental attributes.

**Methods:**

The MCDA process included 1) selection of decisions makers who were representative of those making reimbursement decisions in a specific country; 2) two pre-workshop questionnaires to identify the most important attributes and their relative importance for a positive recommendation for a new drug; 3) a 1-day workshop during which participants undertook three tasks: i) they agreed on a final list of decision attributes and their importance weights, ii) they developed level descriptions for these attributes and mapped each attribute level to a value function, and iii) they developed profiles for hypothetical products ‘just likely to be reimbursed’; and 4) use of the data from the workshop to develop a prediction algorithm based on a logistic regression analysis. The MCDA process is illustrated using case studies for three countries, the United Kingdom, Germany, and Spain. The extent to which the prediction algorithms for each country captured the decision processes for the workshop participants in our case studies was tested using a post-meeting questionnaire that asked the participants to make recommendations for a set of hypothetical products.

**Results:**

The data collected in the case study workshops resulted in a prediction algorithm: 1) for the United Kingdom, the probability of a positive recommendation for different ranges of cost-effectiveness ratios; 2) for Spain, the probability of a positive recommendation at the national and regional levels; and 3) for Germany, the probability of a determination of clinical benefit. The results from the post-meeting questionnaire revealed a high predictive value for the algorithm developed using MCDA.

**Conclusions:**

Prediction algorithms developed using MCDA could be used by pharmaceutical companies when designing their clinical development programs to estimate the likelihood of a favourable reimbursement recommendation for different product profiles and for different positions in the treatment pathway.

The value of a new health technology can be described by a set of product attributes relating to its efficacy, safety, impact on quality of life (QOL) and functional status, dosing convenience, and pricing, compared with the current standard of care (SOC). The achievable price, as well as reimbursement and uptake of the health technology, depend on these product attributes, the severity of the indicated disease, and the unmet need in the indication. The relative importance of the different attributes is different for different decision makers (patients, physicians, and payers), as well as in different countries, and it drives pricing, reimbursement, and market uptake.

In health systems throughout the world, health technology assessment (HTA) plays an essential role in supporting decision making about access to technology, its diffusion, and its innovation. However, huge variation exists regarding defining the value of a new technology, as well as the value criteria, metrics, and assessment processes, depending on how health care is funded in each jurisdiction and the societal consensus and perspective on the value of health.

There continues to be a discussion around the optimal methodology to be used to capture all the criteria that determine the value of a new health care technology. Multicriteria decision analysis (MCDA) has been proposed as an appropriate technique to support decision makers when the assessment of value is complex (1). For example, MCDA has been recommended for the quantification of value of a new drug including both benefits and risks as an input to regulatory decision making (2, 3). Whereas its use in the decision-making process of the National Institute for Health and Care Excellence (NICE) for HTA appraisals is controversial in the United Kingdom (UK) (1, 4), MCDA may be an appropriate tool for manufacturers to include in their market access strategic-planning process.

For a pharmaceutical company developing a new drug, both a qualitative and quantitative understanding of the relative importance of the different product value attributes to the different health care decision makers can help in portfolio investment decisions, clinical development plan design, and marketing strategy development. In this article, we present an MCDA process, using three case study examples, for developing an algorithm that can be used by pharmaceutical companies to predict the likelihood of a new drug achieving a favourable reimbursement recommendation based on actual or expected drug attributes and disease environmental factors.

## Background on MCDA

MCDA is a technique that has been used to compare, prioritise, and select from among alternative strategies or products in situations where many criteria could influence the decision and trade-off need to be made. The technique has been used extensively in public and private sector settings, including health care systems (5–7). A manual describing the different methods, the process, and its use for UK public sector decision makers was published in 2009 (8).

MCDA requires the setting of explicit decision objectives by the decision makers and the definition of a set of measurable criteria that can be assessed for each option being considered and that are relevant to achieve the decision objectives. A formal elicitation process is then followed to estimate the relative weights and values of the different attributes and attribute levels, which allow an overall score to be estimated for each of the options being considered. The results of this process can then be used in various ways to rank the alternative options being considered in an HTA (9). The incorporation of MCDA into HTA for new medical technologies to allow for consideration of a broad range of technology and environmental factors has been proposed by Rotter et al. (10) and Poulin et al. (11).

Although the relative values estimated using an MCDA elicitation process reflect the subjective judgment of the decision makers, this mirrors the decision-making process and so can provide a useful indication of what the actual decision would be, given a set of product attributes (12). Thus, as well as being a tool to ensure that HTA reflects a broad set of technology and environmental factors, MCDA can also be used by a pharmaceutical company to predict reimbursement decisions based on the attributes of a new technology and the environmental factors. It is this latter use that is the focus of this article.

## Methods

### Overview

To use MCDA to generate an algorithm for predicting the probability of a positive reimbursement recommendation from the HTA process for a given market environment and drug indication, a group of health care decision makers should be selected, a set of decision objectives defined, the market and drug attributes of importance for making the decisions identified, and values for the levels of each attribute and the relative importance across the attributes estimated. Because the attributes used for pricing and reimbursements and the relative value of those attributes vary from market to market, they must be assessed in each market separately. To illustrate the proposed MCDA process for generating a prediction algorithm, we present the process that was followed and the results for a case study for three European countries. The countries were the UK, Germany, and Spain, reflecting different types of European health care systems with centralised and decentralised structures.

### Country-specific HTA processes

In the UK, decisions on the adoption of new technologies by the National Health Service (NHS) are primarily based on their incremental cost-effectiveness ratio (ICER), which measures effectiveness in quality-adjusted life-years (QALYs) gained. However, since the Department of Health's decision to use a value-based pricing approach for the valuation of new health care technologies, the paradigm of using the ICER as the only value metric has been recently shifting toward considering other criteria such as equity, innovation, and affordability.

In Germany, the German Act on the Reform of the Market for Medicinal Products (AMNOG), effective since January 2011, has changed considerably the market access of new pharmaceuticals by linking the pricing of pharmaceuticals to the additional benefit of the new drug compared with existing treatment options. The formal assessment by the Institute of Quality and Efficiency in Health Care (IQWIG) is based on the comparative effectiveness of the new pharmaceutical in its indication. The benefit is measured on six different levels: 1) major added benefit over comparator, 2) significant added benefit, 3) slight added benefit, 4) unquantifiable added benefit, 5) no added benefit proven, and 6) less benefit than comparator. The clinical value of the new drug measured by patient-relevant outcomes such as morbidity, mortality, and QOL is the primary value driver for the Joint Federal Committee's (GBA) decision process. Although the consideration of health economic measures and methods has been widely discussed in Germany, cost-effectiveness does not yet play a key role in the GBA's benefit assessment.

In Spain, with the Law of Guarantees and Rational Use of Pharmaceuticals and Health Products, effective since July 2006, the health care system was completely decentralised. Reimbursement and pricing decisions are made on national and regional levels. On the national level, the Ministry of Health decides upon the inclusion of a new pharmaceutical on the national reimbursement list based on data on the duration of adverse events (AEs) related to the drug, the specific needs of some social groups, the therapeutic value of the new drug, cost, existing therapeutic alternatives, the expected market penetration of the new drug, the average market prices of other European Union countries, and the therapeutic utility of the different alternatives, which have to be submitted by the manufacturer. The inclusion of a new pharmaceutical on a regional reimbursement list is decided autonomously by the individual regional authority (13, 14).

### The MCDA process

**Fig. 1.**
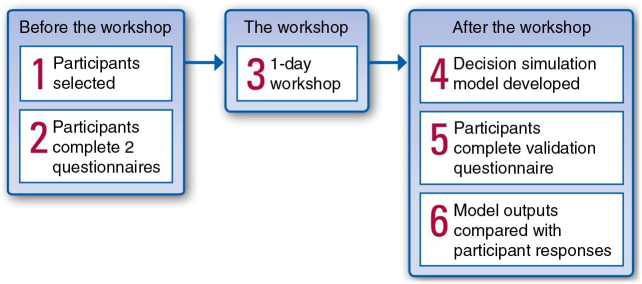
Multicriteria decision analysis process overview.

At the centre of the MCDA process was a facilitated workshop, with tasks undertaken by the participants before the workshop and analysis of the data collected after the workshop. The facilitated workshop took place over 1 day. The participants included decision makers who were typical of those making reimbursement decisions in the market of interest and who were experienced in the reimbursement procedures in that country. The overall process is described in Fig. 1.

For the case study countries, the following steps were completed to derive quantitative estimates of the probability of a positive recommendation for reimbursement for a new drug in the UK, Germany, and Spain.

#### Step 1: recruitment of payers

A list of national payers or payer advisors, including health economists familiar with the practices of the NHS for England and Wales and for Scotland, the Statutory Health Insurances in Germany, and the Spanish national and regional health care systems, was generated by the study sponsor. An initial wave of invitations to persons on the list identified a few participants and allowed dates for the workshops to be scheduled. Additional persons from the list were invited to participate in the case study until the desired number of workshop attendees was reached. Overall, seven decision makers and advisors attended from the UK (of 13 invited), six from Germany (of 16 invited), and eight from Spain (of 11 invited).

#### Step 2: pre-workshop questionnaires

Two pre-workshop questionnaires were sent to the participants of each country to identify the most important attributes and their relative importance for a reimbursement recommendation for a new treatment for a hypothetical but not named chronic non–life-threatening disease.

The first questionnaire included a ‘long list’ of potential reimbursement criteria prepared using information taken from a wide range of sources, including HTAs from reimbursement authorities, criteria identified by Devlin and Parkin (15) for the UK influencing NICE decisions, and criteria relevant to drive decisions of the Pharmaceutical Benefits Advisory Committee in Australia (16). Participants were asked to consider that a new pharmaceutical product had just been approved in their country and that they were members of a committee responsible for deciding on pricing, reimbursement, and/or adoption of the new product. On this basis, participants were asked to select up to 10 attributes they considered highly important for the decision and to identify the three most important attributes from among them.

On the basis of the responses, country-specific short lists (i.e., the highest scoring reimbursement-relevant attributes) were prepared and sent to the participants in a second questionnaire. In this questionnaire, participants were asked to allocate 100 ‘importance points’ among the subset of reimbursement-relevant attributes to reflect the relative importance of the attributes in deciding whether or not the new pharmaceutical product should be reimbursed. The point allocations were then used to calculate importance weights for the reimbursement-relevant attributes for each country.

Although the pre-workshop questionnaires were not pilot-tested before use, they were developed using published data on important attributes, and the participants were allowed to add additional attributes they felt were important. In addition, the purpose of the pre-workshop questionnaires and the participant responses were presented at the beginning of the workshop, and participants were given an opportunity to confirm or change the list of attributes and the values of the importance weights assigned to each attribute.

#### Step 3: decision workshop

A 1-day workshop was held for the participants from each country. After an initial general discussion of the goals for the workshop and the decision-making process in the participants’ own country, three tasks were undertaken.

First, participants confirmed the reimbursement-relevant attributes and, if appropriate, revised the importance weights previously assigned for the key attributes in the second pre-workshop questionnaire. For the UK, based on the pre-workshop questionnaire responses and discussion at the beginning of the workshop, it was agreed that the cost per QALY was the most important attribute and that the relative importance for the other attributes should be assessed for products falling within four different cost-per-QALY ranges, defined as less than £20,000; £20,000–30,000; £30,000–50,000; and greater than £50,000. The relative importance weights for the other attributes for these four cost-per-QALY ranges were then agreed by the workshop participants, although in this article we only present the process and results for the £20,000–30,000 range.

For Germany, participants agreed that the key outcome of the predictive model would be to determine whether the new drug provides additional clinical benefit according to the AMNOG law. Thus, the participants agreed on the relative importance of the nine attributes identified in the pre-workshop questionnaire for a determination of an additional benefit (or not) for a product.

For Spain, based on the general discussion at the beginning of the workshop, two pricing–reimbursement decisions to be modelled were defined by the participants – ‘reimbursement at the central level’ and ‘reimbursement without restrictions at the regional level’ – and the relative importance of the identified attributes was agreed on separately for products being positively recommended for reimbursement on a national and on a regional level.

Second, after agreement on the importance weights for the selected reimbursement attributes, the attributes were divided among the participants, working in pairs, and they were asked to develop level descriptors for these attributes and to assign to each attribute level a value on a scale from 0 to 1 representing the extent to which that attribute level would have a positive impact on the reimbursement recommendation, with 1 indicating the greatest positive impact. Example best and worst attribute-level descriptors, along with the instruction to identify a few intermediate levels for each attribute, were provided to the participants to guide their development of the attribute levels. However, they were told that they could change the best and worst descriptors if desired. A graphical template was also provided to the participants for each attribute, instructing them to place their chosen attribute-level descriptors on the x-axis and plot a value between 0 and 1 using the scale on the y-axis, which represented the impact of each attribute level on a positive reimbursement recommendation. The attribute levels and associated values developed by each pair of participants were then presented to all workshop participants for discussion and agreement.

Third, participants, working in pairs, were asked to develop four ‘marginal profiles’ – listings of attribute levels of each of the product attributes agreed on earlier in the workshop – of a hypothetical new drug that was ‘just acceptable’ for a recommendation of reimbursement. For Spain, participants were asked to also discriminate between national and regional decisions.

#### Step 4: development of prediction algorithms

Separate prediction algorithms were created for each country. The attribute value function scores and the attribute relative importance weights were used to calculate a multi-attribute value score (MVS) for each marginal profile developed during the workshop, that is, MVS=Σw_i_v_ij_, where *MVS* was equal to the sum over all *i* attributes, *w*_*i*_ is the importance weight assigned to the *i*th attribute, *j* is the level selected to represent attribute *i* in the marginal profile, and *v*_*ij*_ is the value assigned to level *j* for attribute *i*. MVS values were transformed to a scale from 1 to 1,000.

The designation of a hypothetical drug profile and its value score as ‘just acceptable for reimbursement’ by the workshop participants was assumed to imply that all lower scores were not acceptable for reimbursement and that all higher scores were acceptable.

A database was created for each marginal profile generated during the workshop with two variables and 1,000 data points. Variable 1 comprised consecutive MVS values from 1 to 1,000, and variable 2 was a dummy variable indicating a positive recommendation for reimbursement (UK) or additional clinical benefit (Germany) (value =1) or not (value =0), depending on the value of variable 1 (IF variable 1<MVS value for marginal profile, THEN variable 2=0, ELSE variable 2=1). The databases for all marginal profiles were combined into one database. Additionally, for the UK, the prediction algorithm incorporated a set of 11 IF…THEN statements to take account of ‘fatalities’ identified by workshop participants during the initial general discussion (e.g., IF ‘ICER £30,000–50,000’ AND ‘AE burden of the new treatment is clinically significantly worse than the SOC’, THEN ‘Not recommended for use in the NHS’).

The MVS databases were used to build a prediction algorithm using logistic regression (plus the IF…THEN statements for the UK) to estimate the probability of a positive recommendation for reimbursement in the NHS for the UK, the probability for a positive assessment of additional clinical benefit in Germany, and the probability of reimbursement on a national and a regional level in Spain as a function of the MVS value. The logistic regressions were carried out using the internet-based program supplied by Pezzullo and Sullivan (17). This is a JavaScript implementation of a standard iterative method to maximise the log likelihood function using Newton's method, with a simple elimination algorithm to invert and solve the simultaneous equations.

#### Step 5: post-workshop internal validation of model outputs

Participants were sent a third questionnaire after the workshop. These country-specific questionnaires comprised 10 product profiles generated using the four most important reimbursement-relevant attributes identified during the workshops for that county and using an orthogonal design such as would be used in a conjoint exercise. Participants were asked to make judgments as to the probability of reimbursement (Spain), additional benefit (Germany), or NHS acceptability (UK) for each profile. These judgments were compared with the predicted probability outputs from the country-specific models to assess the extent to which the prediction algorithms captured the decision-making processes for the workshop participants. The comparisons were estimated using several measures of predictive value: sensitivity, specificity, positive predictive value, negative predictive value, and overall agreement.

## Results

For the case study, from a list of 27 possible attributes, participants in the German, Spanish, and UK workshops identified up to 10 most important attributes for a pricing–reimbursement recommendation for a new drug for treatment of a chronic non–life-threatening disease (see Tables (1–3)).

**Table:** *Table 1*. Attributes and relative importance weights with a cost per QALY of £20,000–30,000 in the United Kingdom

Attribute	Relative importance weight (%)

Robustness of supporting clinical evidence	31
Robustness of modelled ICER	25
Relative efficacy	8
Availability of alternative treatments	8
Relative safety of new drug	7
Ease of adoption of new treatment	7
Incremental impact on quality of life	5
Budget impact	4
Unmet need	3
Size of proposed population	1

ICER=incremental cost-effectiveness ratio; QALY=quality-adjusted life-year.

**Table:** *Table 2*. Attributes and relative importance weights for a determination of additional clinical benefit in Germany

Attribute	Relative importance weight (%)

Robustness of clinical evidence	30
Incremental efficacy	17
Safety of new drug	12
Availability of alternative treatments	10
Unmet need	9
Incremental impact on quality of life	7
Burden of disease	6
Budget impact	5
Availability of other-country evaluations	4

**Table:** *Table 3*. Attributes and relative importance weights for a determination of central and regional reimbursement in Spain

Attribute	Relative importance weight, central level (%)	Relative importance weight, regional level (%)

Incremental efficacy and effectiveness	22	17
Incremental costs and budget impact	16	15
Reimbursed price level of alternative treatments	14	14
Size of proposed reimbursement population	13	13
Availability of alternative treatments	11	12
Safety of the new treatment	9	7
Robustness of the supporting clinical evidence	8	10
Incremental cost-effectiveness ratio	4	7
Ease of adoption of new treatment	2	3
Burden of disease	1	2

For the UK, of those 10 important attributes, the robustness of the supporting clinical evidence and the robustness of the modelled ICER were assigned the highest weights for a positive recommendation for reimbursement (Table 1). For Germany, the three most important attributes were the robustness of the supporting clinical evidence, the incremental efficacy of the new drug, and the safety of the new drug (Table 2). For Spain, the three most important attributes for both national and regional decision makers were incremental effectiveness, incremental cost-effectiveness and budget impact (BI), and reimbursement price level of alternative treatments (Table 3). The incremental effectiveness was slightly more important for national than for regional decision makers.

Participants defined up to six levels for each attribute and assigned relative values on a scale from 0 to 1 for a positive recommendation for reimbursement. For the attribute ‘robustness of the clinical evidence’, for example, the following attribute levels were defined by the participants from the UK:

- Level 1: clinical evidence not relevant to payers
- Level 2: weak intermediate endpoints and indirect comparisons
- Level 3: relevant endpoints and comparators

For the same attribute, participants from Germany defined the following levels:

- Level 1: Endpoints or comparators not relevant to patients
- Level 2: Clinical endpoints relevant but not comparators
- Level 3: Clinical endpoints and comparators relevant

For the same attribute, participants from Spain defined the following levels:

- Level 1: Low quality/quantity of data; comparator(s) of little relevance to payers
- Level 2: Evidence based on intermediate endpoints that are not considered robust
- Level 3: Evidence based on robust endpoints, with external validity against current practice
- Level 4: Evidence based on robust endpoints, high quality and quantity of data, and published in peer-reviewed journals

**Fig. 2.**
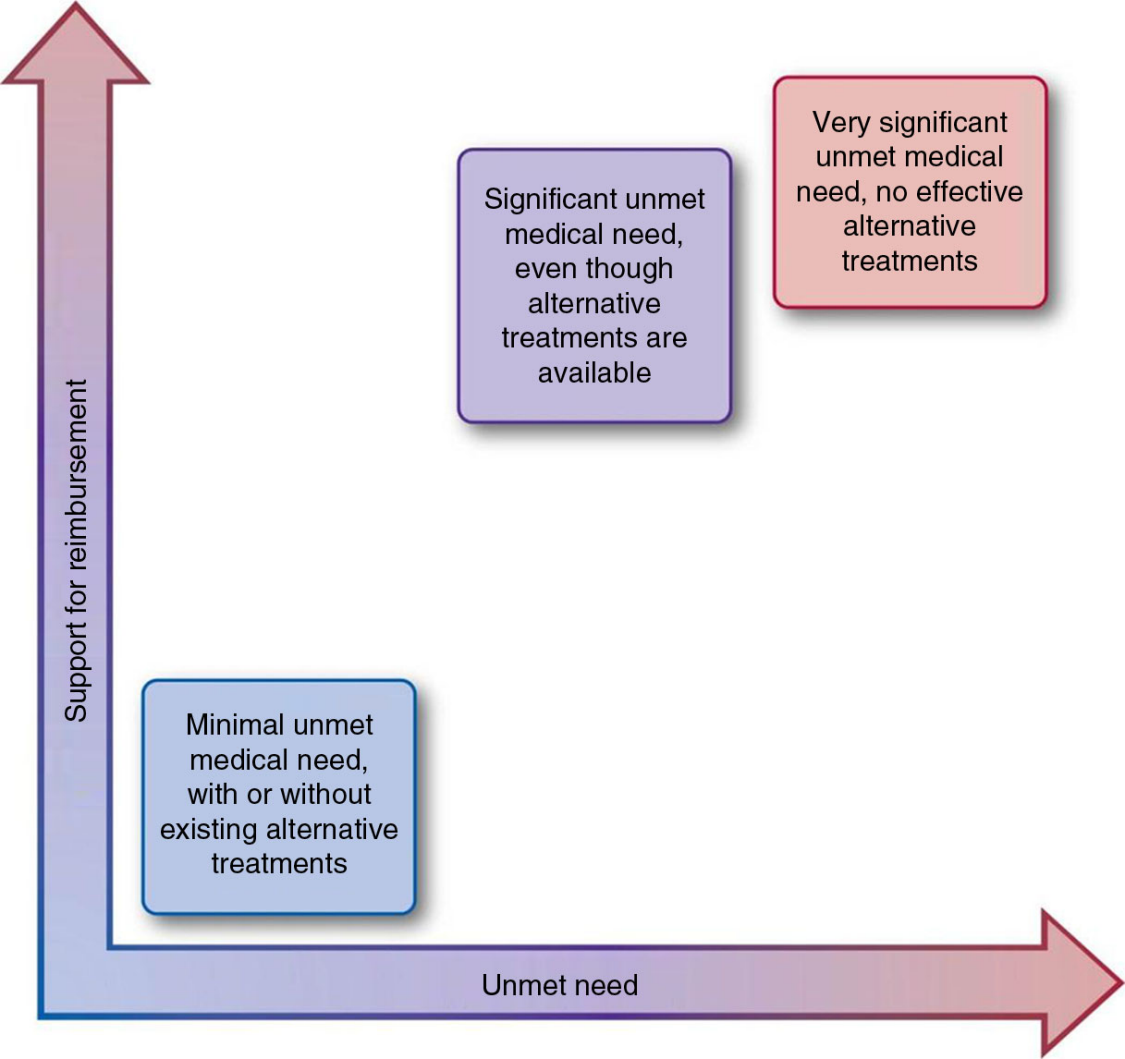
Creation of a value function (‘unmet need’ attribute).

The shape of the value function for unmet need is illustrated in Fig. 2.

Table [Table T0004], 5 [Table T0005], and 6 present all levels for the up to 10 most important attributes and their relative values on a scale from 0 to 1. The lower the value, the less supportive the evidence of this attribute would be for a positive reimbursement recommendation.

Logistic regression equations were estimated based on the data collected during the decision workshops (Table 7).

These regression equations were used to estimate the probability of a positive recommendation for reimbursement (UK), a favourable assessment of additional clinical benefit (Germany), or a positive reimbursement outcome on a national and a regional level in Spain of a hypothetical new product (P_new_). First, the product was rated on the attributes by assigning levels and using these ratings with the importance weights agreed during the workshop to calculate an MVS for the new product (MVS_new_).

For the UK, the MVS_new_ for the new product was entered into the following equation to estimate the probability of a positive reimbursement recommendation for a new product with an estimated cost per QALY between £20,000 and £30,000:

$$Pnew=\frac{1}{1+e^{-6.2354+0.0101\times MVSnew}}$$

For Germany, the MVS_new_ was entered into this equation to estimate the probability of a favourable additional clinical benefit:

$$Pnew=\frac{1}{1+e^{-7.6134+0.0157\times MVSnew}}$$

For Spain (national), the MVS_new_ was entered into this equation to estimate the probability of a positive reimbursement outcome:

$$Pnew=\frac{1}{1+e^{-14.9559+0.0352\times MVSnew}}$$

For the regional model for Spain, the MVS_new_ was entered into this equation:

$$Pnew=\frac{1}{1+e^{-10.6314+0.0244\times MVSnew}}$$

The results of the internal validation exercises using data from the post-meeting questionnaire are shown in Table 8.

**Table T0004:** *Table 4*. Attribute levels and relative values for a positive recommendation for reimbursement in the United Kingdom

Attribute	Level 1 value	Level 2 value	Level 3 value	Level 4 value

Robustness of supporting clinical evidence	0 Clinical evidence not relevant to payers	0.25 Weak intermediate endpoints and indirect comparisons	1 Relevant endpoints and comparators	
Robustness of modelled ICER	0 Model structurally invalid	0.52 De novo model with no validation and limited data for input values	1 Well-established model, strong input data sources, and SOC comparator	
Relative efficacy	0 Inferior to SOC	0.31 Equivalent to SOC	0.63 Marginally superior to SOC	1 Markedly superior to SOC
Availability of alternative treatments	0 >3 differentiated alternative treatments	0.36 1–3 differentiated alternative treatments	1 No effective alternative treatments	
Relative safety of new drug	0 AEs worse than SOC	0.65 AEs same as SOC	1 AEs better than SOC	
Ease of adoption of new treatment	0 Major changes in service delivery	0.71 Unclear whether service delivery will change	1 No changes in service delivery	
Incremental impact on quality of life (using standard scale)	0 Utility score worse by ≥10%	0.71 Some improvement in utility	1 Improvement in utility ≥30%	
Budget impact	0 Increase in total health care costs	0.5 Total health care costs do not change	1 Decrease in total health care costs	
Unmet need	0 Lifetime reduction <0.3 QALYs	1 Lifetime reduction 0.3–3 QALYs	1 Lifetime reduction >3 QALYs	
Size of proposed population	0 >200,000	0.36 7,000–200,000	1 <7,000	

AEs = adverse events; ICER = incremental cost-effectiveness ratio; QALYs = quality-adjusted life-years; SOC = standard of care.

Values between 0 and 1, with lower values representing a lower level of support for a favourable reimbursement recommendation.

**Table T0005:** *Table 5*. Attribute levels and relative values for a favourable assessment in Germany

Attribute	Level 1 value	Level 2 value	Level 3 value	Level 4 value	Level 5 value

Robustness of clinical evidence	0 Endpoints or comparators not relevant to patients	0.76 Clinical endpoints are relevant, but comparators are not	1 Clinical endpoints and comparators relevant		
Incremental efficacy	0 Inferior to SOC	0.04 Inferior to best available treatment	0.30 Superior to SOC on robust surrogate outcomes	0.82 Superior to SOC on patient-relevant outcomes	1 Superior to best available treatment in head-to-head trials
Safety of new drug	0 AEs worse than SOC	0.45 AEs same as SOC	1 AEs better than SOC		
Availability of alternative treatments	0 At least 1 alternative treatment	1 No alternative treatment			
Unmet need	0 Minimal	0.83 Significant despite available treatments	1 Significant; no effective treatments		
Impact on QOL	0 No evidence of impact on QOL	0.35 Indirect evidence of QOL benefit	1 QOL benefit in direct comparison with SOC		
Burden of disease	0 Minimal impact on activity and cost	1 Large impact on activity and high cost			
Budget impact	0 Increase in total health care costs	0.86 Total health care costs do not change	1 Decrease in total health care costs		
Availability of other-country evaluations	0 None available	0.19 Available, but flawed or not relevant for Germany	1 Available, good quality, and relevant for Germany		

AEs=adverse events; QOL=quality of life; SOC=standard of care.

Values between 0 and 1, with lower values representing a lower level of support for a favourable reimbursement recommendation.

**Table:** *Table 6*. Attribute levels and relative values for a favourable assessment in Spain (national level)

Attribute	Level 1 value	Level 2 value	Level 3 value	Level 4 value	Level 5 value	Level 6 value

Size of proposed reimbursement population	0 High incidence/prevalence	0.16 Intermediate incidence/prevalence	0.24 Low incidence/prevalence	1 Orphan-size population		
Burden of disease	0 Minimal societal burden	0.33 Low societal burden	0.66 High societal burden and low impact on patient	1 High societal burden and high impact on patient		
Availability of alternative treatments	0 Other drugs in the same class available	0.3 Other drugs available but different class	1 No other drugs available			
Reimbursed price level of alternative treatments	0 Price >20× price of drug to be replaced	0.18 Price >10× price of drug to be replaced	0.36 Price >5× price of drug to be replaced	0.80 Price equal to price of drug to be replaced	1 Price less than price of drug to be replaced	
Incremental efficacy and effectiveness	0 Inferior efficacy on clinically relevant endpoints	0.44 Equivalent efficacy on clinically relevant endpoints	1 Superior efficacy on clinically relevant endpoints			
Robustness of the supporting clinical evidence	0 Low quality and little relevance to payers	0.03 Intermediate endpoints; not robust	0.51 Robust endpoints with external validity	1 Robust endpoints, high-quality data, and published in peer-reviewed journals		
Safety of the new treatment	0 Low safety; high incidence of AEs versus SOC	0.37 No difference from SOC	1 Greater safety and increased compliance versus SOC			
Incremental costs and budget impact	0 Costs ~100% > SOC	0 Costs ~50% > SOC	0 Costs ~20% > SOC	0.52 Cost neutral	0.63 Cost savings	1 Budget savings
Ease of adoption of new treatment	0 No guidelines; new processes required	0.33 Clear guidelines; treatment processes not well defined	1 Guidelines available no uptake barriers			
Incremental cost-effectiveness ratio	0 ICER much greater than societal WTP	0.55 ICER greater than societal WTP	1 ICER much less than societal WTP			

AEs=adverse events; BI=budget impact; ICER=incremental cost-effectiveness ratio; SOC=standard of care; WTP=willingness to pay.

Values between 0 and 1, with lower values representing a lower level of support for a favourable reimbursement recommendation.

**Table:** *Table 7*. Logistic regression equations by country

Country	Logistic regression equation

Germany	Y=−7.61364+0.0157×MVS
Spain (national)	Y=−14.9559+0.0352×MVS
Spain (regional)	Y=−10.6314+0.0244×MVS
United Kingdom	Y=−6.2354+0.0101×MVS

Y is the log-odds of a positive recommendation for reimbursement in the National Health Service (in the United Kingdom), a favourable assessment of additional clinical benefit (Germany), or a positive reimbursement decision (Spain); MVS is the multi-attribute value score as described in the “Methods” section.

**Table:** *Table 8*. Validation indices

Validation	Explanation	Germany	Spain	United Kingdom	All three countries

Sensitivity	The probability that a positive decision as assessed by the post-workshop questionnaire will be accurately identified by the model	71% (10/14)	71% (24/34)	71% (5/7)	71% (39/55)
Specificity	The probability that a negative decision as assessed by the post-workshop questionnaire will be accurately identified by the model	85% (22/26)	100% (6/6)	91% (21/23)	89% (49/55)
Positive predictive value	The probability that a positive decision from the model also will be positive as assessed by the post-workshop questionnaire	71% (10/14)	100% (24/24)	71% (5/7)	87% (39/45)
Negative predictive value	The probability that a negative decision from the model also will be negative as assessed by the post-workshop questionnaire	85% (22/26)	38% (6/16)	91% (21/23)	75% (49/65)
Overall agreement	The probability of agreement between the model and post-workshop questionnaire decisions (positive and negative taken together)	80% (32/40)	77% (30/40)	87% (26/30)	80% (88/110)

## Discussion

The aim of this article was to present an MCDA process to develop an algorithm for a pharmaceutical company to use for predicting pricing and reimbursement recommendations by HTA agencies for new drugs. Our case study resulted in prediction algorithms for the probability of a positive reimbursement recommendation by NICE in the UK, a favourable additional clinical benefit assessment by the GBA in Germany, or a positive reimbursement outcome nationally or regionally in Spain for a new product in development. The process included eliciting the most important product attributes and their relative importance to reimbursement decision makers for their recommendations or decisions through pre-workshop questionnaires and in a face-to-face workshop, with internal validation on the quantitative findings using the estimated model and a post-workshop questionnaire. In our case study, the results of the MCDA process provided qualitative information through the taped workshop discussions as well as prediction algorithms that could be used to estimate the probability of a favourable assessment of a new drug by reimbursement decision makers based on actual or expected drug and environmental attributes.

During the three country-specific decision workshops, which were face-to-face meetings (with seven participants from the UK, six from Germany, and eight from Spain), the general discussion at the beginning of the workshop was found to be critical for ensuring that the subsequent valuation exercises were targeted to the actual decision-making process in that country. For example, for the UK, after the initial general discussion, the participants agreed that rather than include the cost-effectiveness ratio as one of the decision criteria, it should be used to categorise potential submissions to NICE or the Scottish Medicines Consortium by ICER ranges before going through the importance weights, valuation, and trade-off exercises for the other product attributes. For the German workshop, the general discussion ended with agreement that the decision being modelled was not a reimbursement recommendation per se but additional clinical benefit. And, for the Spanish workshop, the participants agreed that there were different importance weights, valuation weights, and trade-offs for central and regional decisions.

For the case study presented in this article, the MCDA process and results were demonstrated for drugs for any chronic non–life-threatening condition rather than for a specific drug or for a specific disease. Although not reported, the same process was completed at the same time for three other types of diseases (chronic life-threatening, acute non–life-threatening, and acute life-threatening) with similar results. Disease types rather than specific diseases were used because the MCDA process is complex and needs to be undertaken in each country separately, and it was important to develop a prediction algorithm that could be applied across a range of diseases and drugs for each country. Clinical experts in a disease area could apply the prediction algorithm to a specific drug and disease indication by mapping the drug, disease, and environment attributes to the attribute descriptors used in the model for each country. For example, for a multiple sclerosis drug, the definitions of clinical effectiveness relative to SOC (marginally superior or markedly superior) could be quantified by the clinical experts in terms of the magnitude of the impact of the drug on disease progression. Thus, we believe that these prediction algorithms developed for different hypothetical disease types or ranges of cost-effectiveness ratios could be used to estimate the probability of favourable reimbursement recommendations across a broad range of drugs and diseases.

Our model findings were tested for internal validation by using a post-meeting questionnaire, which revealed a high predictive value of the MCDA models compared to workshop participant responses to questions about the probability of reimbursement for products with a range of attribute levels for all three countries. This shows that a prediction algorithm using an MCDA process could be a useful tool to support market access strategies for new drugs early in development. It provides information to pharmaceutical companies for anticipating reimbursement decisions that extends the insights gained from traditional qualitative payer research, because the reasons for the payer decisions are more transparent. The method can be applied in different countries by simulating the decision process used in each country in the elicitation exercises. This allows decision criteria specific to each country and relevant to the disease area of interest to be more systematically recognised and applied. Scores for the relative value of each attribute level and the relative importance weights for the attributes for the pricing and reimbursement decisions are elicited using formal and established techniques. The performance of current treatments on the attributes can be obtained from credible external sources (e.g., clinical trial results and published economic models). Information on the performance and attributes of the new product can be obtained from early clinical trial data and early economic modelling.

The prediction algorithm generated using an MCDA process could be used for early pricing strategies; design of drug development plans, including clinical trial design and choice of position in the treatment pathway; and market access planning to minimise the uncertainty around payer value assessments of a new drug in development. The market value of different positioning options of a new compound could be tested from a reimbursement perspective. Phase 3 evidence plans could be informed, and their probability of success in terms of market access could be estimated. The application of prediction algorithms could also help to inform pricing strategies and support targeted value messages that are based on the comparative benefit defined by the product attributes and value drivers relevant to payers. The trade-offs and related opportunity costs for development decisions can be better understood and make the risk and uncertainty around investment decisions more transparent.

Our internal validation approach, of providing a series of drug profiles likely to lead to different reimbursement decisions and sending it to the workshop participants in a post-workshop questionnaire, provides a test of internal validation, the extent to which our prediction algorithm captured the decision processes of the workshop participants. This task had to be completed after the workshop, because the hypothetical drug profiles were developed using the importance weights and levels determined during the workshop. A more robust external test of the validity of the prediction algorithms would be to apply the prediction algorithms to recent decisions for drugs for different types of disease. Such a test would require mapping the attribute levels for each drug indication into the generic attribute descriptors. This could be an interesting topic for future research.

It is important to bear in mind the limitations of the approach. The prediction algorithm is based on the judgments of a limited number of people; their views may be inconsistent and may easily change over time. Other similar people may hold different views, and real-life decisions are influenced by issues not considered in the models (e.g., politics and personalities). Also, other approaches for the use of the data generated using the MCDA process exist (9), but none of them have ever been thoroughly tested to assess the value of health care interventions from a payer's perspective. Although the attributes vary between different health care systems, the selection within a system should meet certain conditions to increase the credibility of the MCDA process. This would include relevant, complete, non-redundant, feasible, and understandable attributes that are clearly defined, judgmentally independent, and scalable (1). The meaning of the weights should be clearly defined in terms of the trade-offs between the selected attributes (1). The method by which we allowed the workshop participants to select the up to 10 most important attributes did not ensure that these conditions were met. Another limitation is related to the fact that health care systems are dynamic systems where reimbursement-relevant attributes may change over time and become either more or less relevant. Finally, use of the MCDA process described in this article was limited to development of an algorithm for predicting reimbursement decisions for use by a pharmaceutical company to inform drug development and market access strategies. Thus, the results would not provide guidance to a group of individuals making an actual decision such as one that would be provided by the results of the MCDA proposed by Walker et al. (2) or Mussen et al. (3) for the quantitative benefit–risk assessment of new drugs.

As with all models, prediction algorithms of this kind should be used only as a guide. Thus, they should be used as decision support and as a starting point for discussion rather than as the sole basis for internal decision making about drug development and marketing strategy. Further research is required to test different MCDA approaches and validate the robustness of the predictive models for different health care systems.
